# Ethereal Pyrotechnics

**Published:** 1867-01

**Authors:** 


*Ethereal Pyrotechnics.*—A correspondent of the *London Lancet*, October 27, relates the following:—

"A gentleman calling to have a tooth extracted, I proceeded in the usual way. Having applied a small piece of cotton-wadding over the tongue, with the view of protecting it from the fluid which I was about to direct upon the upper jaw, I commenced business. I should say that in *every* preceding instance I have used a candle, to throw a better light into the mouth. This is held by the assistant not nearer than half a yard from the seat of operation. In this case it was done also. And now comes the terrible scene. I had scarcely used the ether (pure rectified) for twenty seconds when suddenly a volume of flame rushed from the patient's mouth, enveloping the three of us for a single instant. It was soon over that the patient had not time to rise from his seat, and the assistant and myself remained in our former positions. There was no explosion; all was quiet. After regarding each other for a few moments, I ventured to inquire of the patient how he felt. I was happy to see a smile, rather ghastly nevertheless, illumine his pale countenance; but his only answer was, "What a wonderful occurrence!"

There was no smell of scorching; the only injury sustained being a slight singeing of the more prominent hairs of his moustache. The assistant and myself were untouched. This was certainly an unexpected and terrible occurrence, though fortunately unattended by any untoward result. The patient's complexion was of a healthy, ruddy color on sitting down for operation; but this soon gave place to a ghost-like pallor; and when I beheld the flames gushing forth from the mouth, I almost believed it was a veritable fire-demon sitting before me. He certainly did not look ethereal. The only unpleasant feeling he experienced was a sense of constriction round his neck. He is nothing the worse for it; in fact, better as he has not felt a twinge of toothache since.

"Now, sir, cases like these are not to be made *light* of. The cause of the mischief might be attributed to the candle. If so, then why did the same effects not ensue in preceding instances, as the same precautions were adopted? I should esteem it a favor to be informed if any rules are laid down for operating in gas-light, as till then I shall be obliged to desist."

[This reminds us of a case which occurred a few years since in Kentucky, and which was described to us by the practitioner in attendance. A patient was suffering severely from colic and intestinal obstruction. Ordinary remedies proving of no avail, the practitioner admistered an enema, which contained sulphuric ether. Feeling that he was about to have an evacuation, and there being no other convenience at hand, the patient rushed for the stone hearth in front of an old fashioned fireplace, in which a huge fire was blazing. An enormous discharge of wind was the first to escape, and from the patient's proximity to the fire a pyrotechnic display resulted, which utterly astounded both physician and patient, the hasty retreat of the latter across the room, leaving, like a meteor, a dazzling train behind! Like the case above related, "the patient felt none the worse for it; in fact better," and of his colic he was entirely relieved.]—*Cincinnati Journal of Medicine.*

